# Efficacy and tolerance of second-generation antipsychotics in anorexia nervosa: A systematic scoping review

**DOI:** 10.1371/journal.pone.0278189

**Published:** 2023-03-16

**Authors:** Solène Thorey, Corinne Blanchet, Sélim Benjamin Guessoum, Marie Rose Moro, Maude Ludot, Emilie Carretier

**Affiliations:** 1 APHP, Cochin Hospital, Maison de Solenn, Paris, France; 2 Versailles Saint-Quentin-en-Yvelines University, Versailles, France; 3 UVSQ, Inserm, CESP, Team DevPsy, Paris-Saclay University, Villejuif, France; 4 Laboratoire de Psychologie Clinique, Psychopathologie et Psychanalyse, Paris Cité University, Boulogne-Billancourt, France; University of Catania Libraries and Documentation Centre: Universita degli Studi di Catania, ITALY

## Abstract

**Introduction:**

Second-generation antipsychotics (SGAs) are frequently prescribed for the treatment of resistant anorexia nervosa. However, few clinical trials have been conducted so far and no pharmacological treatment has yet been approved by the Food and Drug Administration. The aim of this paper is to conduct a systematic scoping review exploring the effectiveness and safety of atypical antipsychotics in anorexia nervosa (AN).

**Method:**

We conducted a systematic scoping review of the effectiveness and tolerability of SGAs in the management of AN. We included articles published from January 1, 2000, through September 12, 2022 from the PubMed and PsycInfo databases and a complementary manual search. We selected articles about adolescents and adults treated for AN by four SGAs (risperidone, quetiapine, aripiprazole or olanzapine). This work complies with the Preferred Reporting Items for Systematic Reviews and Meta-Analysis extension for scoping reviews (PRIMA-ScR) and was registered in the Open Science Framework (OSF) repository.

**Results:**

This review included 55 articles: 48 assessing the effectiveness of SGAs in AN and 7 focusing only on their tolerability and safety. Olanzapine is the treatment most frequently prescribed and studied with 7 randomized double-blind controlled trials. Other atypical antipsychotics have been evaluated much less often, such as aripiprazole (no randomized trials), quetiapine (two randomized controlled trials), and risperidone (one randomized controlled trial). These treatments are well tolerated with mild and transient adverse effects in this population at particular somatic risk.

**Discussion:**

Limitations prevent the studies both from reaching conclusive, reliable, robust, and reproducible results and from concluding whether or not SGAs are effective in anorexia nervosa. Nonetheless, they continue to be regularly prescribed in clinical practice. International guidelines suggest that olanzapine and aripiprazole can be interesting in severe or first-line resistant clinical situations.

## Introduction

Anorexia nervosa (AN) is a disorder characterized according to the DSM 5 by a restricted calorie intake aimed at maintaining a low weight, intense fear of gaining weight or being fat, or persistent behavior interfering with weight gain, and disruption of body image (body dysmorphic disorder), associated with a persistent lack of recognition of the seriousness of the current low body weight. It is a relatively frequent chronic disorder with a prevalence of 0.9 to 1.5% among women and 0.2 to 0.3% among men [1].

AN is a serious disease with the highest mortality rate of all mental illnesses (between 6 and 18%, depending on the study) [2] with a high risk of relapse (estimated at about 30%) [3]. It remains poorly understood, associated with numerous comorbidities, and difficult to treat. Currently, treatment for AN is multidisciplinary, based principally on the management of risks and somatic complications together with regular psychotherapy. The US Food & Drug Administration (FDA) has not yet approved any pharmacological treatment for it.

In recent years, various international teams specialized in eating disorders (EDs) have looked at the role of second-generation antipsychotics (SGAs) in the therapeutic arsenal against AN. Several theoretical and clinical observations may justify their use in this indication. First, AN is characterized by an almost delusional conviction that one is overweight associated with an inordinate fear of gaining weight [4]. These certainties cannot be touched by logic or reassurance, and these patients often demonstrate cognitive rigidity. SGA may help to reduce the invasiveness of these false beliefs and diminish the rigidity of the patients’ thoughts [4]. In addition, SGAs and especially olanzapine are associated with a significant weight gain in the different psychiatric populations (children as well as adults) for whom these treatments are usually prescribed [5]. SGAs are also known to have a strong affinity for the serotoninergic, histaminergic, and adrenergic receptors involved in appetite and food intake. These serotoninergic neuronal systems are frequently dysfunctional in AN patients [6,7]. The prescription of an SGA might allow a better regulation of these circuits. Finally, AN is associated with numerous psychiatric comorbidities, especially symptoms of anxiety and obsessiveness as well as mood disorders [8]. Their sedative, anxiolytic [9,10], and mood-regulating effects [11] may enable this drug class to reduce obsessive ideas and anxiety and thus to improve mood stability.

At the end of the 1990s, the first encouraging case reports [12] opened up research into the role of SGA in EDs. Several studies, such as the overview published by Blanchet et al. [13], have looked at the question of pharmacological treatment in AN, but few have concentrated specifically on the evaluation of SGAs in this disorder. Most of the published studies, such as those by Kishi et al. [14] and Lebow et al. [15] do not include many subjects, do not focus on several SGAs and are often limited to adults population. This review is the first to gather such a large number of published articles on this topic in both child and adult populations while considering several SGAs.

The objective of our study was to perform a systematic scoping review of the literature covering all of the studies since the use of SGAs began to develop that have assessed their effectiveness and tolerability in AN management.

## Material and methods

This scoping review of the literature [16,17] on the effectiveness and tolerability of SGAs in the management of people with AN applied the Preferred Reporting Items for Systematic Reviews and MetaAnalyses extension for scoping reviews (PRISMA-ScR) statement [18]. See S1 File. This statement, developed by expert consensus through a Delphi panel, contains a checklist of 20 essential reporting items and 2 optional items, all important in the optimal methodology of all scoping reviews. Adherence to the PRISMA-ScR statement is essential for enhancing the methodological consistency and uptake of research findings across scoping reviews. In contrast to systematic reviews, scoping reviews do not usually provide a critical appraisal of the studies they include. The protocol for this scoping review was registered with the Open Science Framework at: [https://osf.io/9ha2z/?view_only=b2e7c500ee7143cda0b0cfe209633cb1].

- Identification of the research question
  We developed a broad research question for our literature search, asking: *“How does the literature inform us about the effectiveness and tolerability of SGAs among people with AN*?*”* This question is important because psychiatrists have increasingly used SGAs for patients with AN since the turn of the century [19], even though their efficacy and tolerability remain poorly studied in this population.
- Identification of the relevant studies
  We selected the articles meeting the following inclusion criteria: (i) any patient aged 10 years or older, that is, preadolescents, adolescents, and adults, (ii) treated as an outpatient or inpatient (iii) for an ED, specifically a restrictive or mixed AN, (iv) by an SGA (v) without a diagnosis of an associated psychotic disorder, and (vi) published in English or French at any time from 2000 through 2022. We included articles over this period because prescriptions of SGAs began proliferating around 2000 and research on their use in AN began then. Before 2000, very few articles investigated EDs and antipsychotic medications [19], and they were almost exclusively about first-generation antipsychotics (FGA) [20,21]. We found only one study [12] about an SGA (olanzapine) and EDs published before 2000. Because there are few studies on drug treatments for EDs, we decided to apply broad phenotypic criteria. We included a large population (preadolescent, adolescent, and adult), both genders (male and female) and receiving all types of care (inpatient or outpatient). We excluded prescriptions for patients with psychotic disorders together with EDs to be able to focus on their effects on the latter. We selected the four SGAs—aripiprazole, olanzapine, quetiapine, and risperidone—most frequently prescribed in AN, according to Beykloo et al. [22]. We did not apply any exclusion criteria related to the type of publication or the study methodology. As recommended for scoping reviews, we did not use study quality as an inclusion criterion [23]. We performed a systematic literature search in the MEDLINE and PSYCINFO databases from January 1, 2000 through September 12, 2022. We used the following key words: (anorexia nervosa [Title/Abstract]) and (atypical antipsychotic or second generation antipsychotic or quetiapine or olanzapine or aripiprazole or risperidone). Three investigators (EC, ML, and ST) screened the references retrieved for eligibility both at the title/abstract and full-text levels. We used Zotero to manage the collection and deduplication of records. For title and abstract screening, we searched manually. We documented and reported reasons for the exclusion of full texts with Microsoft Excel. Two review authors (EC and ST) extracted and charted study characteristics and data into the categories of the data extraction form in Excel. These categories included a priori categories, based on our initial understanding of SGA prescription for EDs. They included the population (e.g., gender, age group), setting (e.g., type of ED); types of interventions (e.g., SGA dose), and study designs. Disagreements were resolved through consensus with the three senior authors of this study (CB, MRM, and SBG).

## Results

**Fig 1:**
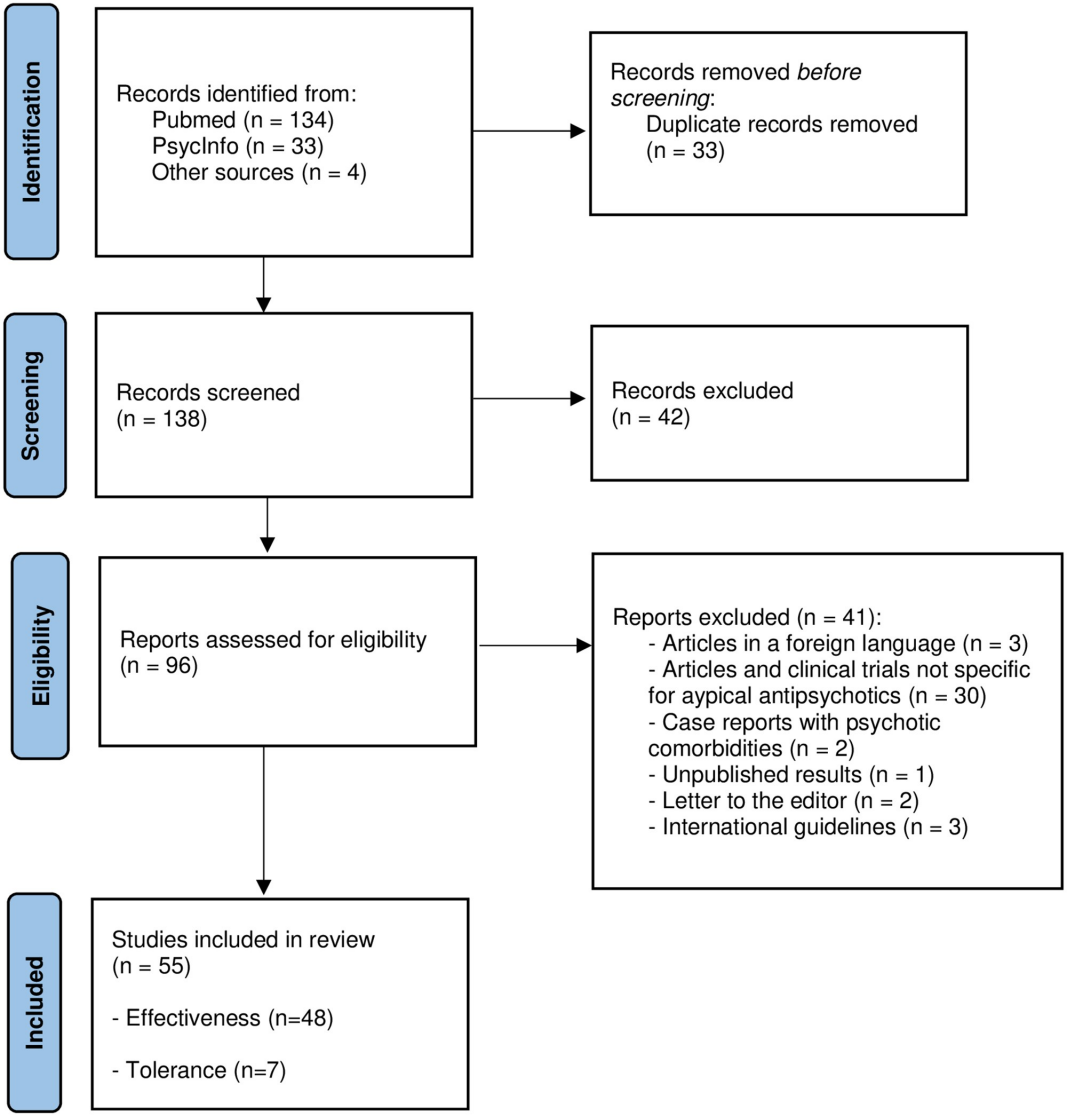
PRISMA 2020 flow chart of the systematic scoping review.

This search produced 167 results. After reviewing the titles, we excluded 75 articles that were not related to the subject under study or were duplicates. A manual search of the bibliographies of the remaining articles and related sources enabled us to add 4 references. Our reading of the abstracts of these 96 articles showed that 55 were eligible, while 41 were excluded on the basis of the relevance criteria defined above. Most of the excluded articles concerned overall pharmacological management of AN and were not limited specifically to SGAs. Reasons for excluding selected studies are summarized in S1 Table (Characteristics of excluded studies). Overall, this literature review includes 55 articles: 20 case reports, 8 retrospective studies, 16 clinical trials (RCTs and open-label), 6 reviews of the literature, and 5 meta-analyses. See Fig 1 (the study flow chart) for additional details. The first part of our results covers the studies concerning the effectiveness of SGAs (48 articles) and the second part those dealing exclusively with their tolerability (7 articles). In each part, the studies are presented according to the particular SGA concerned: olanzapine, aripiprazole, quetiapine, and risperidone.

### I. Effectiveness of SGA in the management of AN

#### a) Olanzapine

In all, 24 of the 48 articles studied olanzapine (Table 1). It is by far the SGA studied most often for AN treatment. It is notably a dopamine D2 antagonist and an inverse agonist of the serotonin 2A and histamine H1 receptors [24]. It is often the treatment of choice among SGAs in light of its known effects on weight and sedation. Moreover, it presents fewer cardiac side effects, has a lower risk of a QT prolongation on ECG, and is associated with fewer extrapyramidal symptoms and less prolactin elevation [25].

**Table 1:** Articles about olanzapine included in the scoping review.

Study	Year	Country	Study method	Number of subjects	Mean age and/or range	Type of ED	AAP + dose and/or mean dose	Study duration	Results
**Attia et al.**	2011	USA	Double-blinded randomized controlled clinical trial	23 (1♂) 11 OLA vs 12 placebo	27.7 years	Anorexia nervosa	Olanzapine 2.5–10 mg/d	8 weeks	BMI significantly higher in the olanzapine than in the placebo group Good clinical tolerance
**Attia et al.**	2019	USA	Double-blinded randomized controlled clinical trial	152 75 OLA vs 77 placebo	28–30 years	Anorexia nervosa	Olanzapine 2.5–10 mg/d DM = olanzapine 7.7 mg/d	16 weeks	Weight gain significantly greater in the olanzapine group No difference between the two groups for obsessive ideas
**Barbarich et al.**	2004	USA	Uncontrolled nonrandomized open-label clinical trial	17	20.5 years	Anorexia nervosa	Olanzapine 4.7 mg/d	6 weeks	Significant difference in weight Reduction in ED symptoms
**Bissada et al.**	2008	Canada	Double-blinded randomized controlled clinical trial	34 18 OLA vs 16 placebo	23.6–29.6 years	Anorexia nervosa (16) Anorexia nervosa with bingeing-purging behavior (18)	Olanzapine 2.5–10 mg/d	13 weeks	Increased weight in both groups but higher in the placebo group
**Boachie et al.**	2003	Canada	Case report	4	10–12 years	Anorexia nervosa (3♀) Unspecified ED (1♂)	Olanzapine 2.5 mg/d	5 weeks in hosp and 3.5 months postdischarge// 1 week before admission, 36 days in hospital, then 4 months postdischarge// 10 weeks in hosp and 2 weeks postdischarge// 52 days in hospital, then 2.5 months postdischarge	Diminution of meal-related anxiety and agitation Improved adherence Weight gain Good tolerance in the general pediatric population
**Bosanac et al.**	2003	Australia	Retrospective study	14	19–49 years	Anorexia nervosa (57%) Anorexia nervosa with purging behavior (43%)	Olanzapine 9.7 mg/d	25 days	Weight gain
**Brambilla et al.**	2007	Italy	Double-blinded randomized controlled clinical trial	20 10 OLA vs 10 placebo	23 years	Anorexia nervosa	Olanzapine 2.5 then 5 mg/d	3 months	No significant difference in weight between the two groups No correlation between ghrelin and leptin levels and weight variations
**Brambilla et al.**	2007	Italy	Double-blinded randomized controlled clinical trial	30	23.7–26.3 years	Anorexia nervosa (18) Anorexia nervosa with bingeing-purging behavior (12)	Olanzapine 2.5 then 5 mg/d	3 months	No significant difference in weight between the two groups Olanzapine may be effective against obsessive ideas
**Dennis et al.**	2006	USA	Case report	5	12, 15, 15, 18 and 18 years	Anorexia nervosa (4♀) Bulimia (1♂)	Olanzapine 2.5–7.5 mg/d	Several months// Several weeks// 6 months// Not reported// Not reported	Improved sleep Diminution of ruminations focused on food and body image Reduced anxiety around meals
**Duvvuri et al.**	2012	USA	Case report	2	12 years (twins)	Anorexia nervosa	Olanzapine 2.5 mg vs *Fluoxetine 60 mg*	36 weeks	Weight gain Remission of symptoms
**Ercan et al.**	2003	Turkey	Case report	1	15 years	Anorexia nervosa	Olanzapine 10 mg/d	28 weeks	Weight gain Remission of symptoms
**Himmerich et al.**	2017	UK	Retrospective study	12	18–60 years	Anorexia nervosa	Olanzapine (dose?)	8 weeks	Significant weight gain on starting olanzapine Good tolerance profile with few modifications in laboratory values
**Jensen et al.**	2000	Denmark	Case report	3	30, 34, and 50 years	Anorexia nervosa AN + borderline personality disorder (1)	Olanzapine 5 mg/d	2 months// 2 months// 9 months	Weight gain Reduction in body distortion
**Kafantaris et al.**	2011	USA	Double-blinded randomized controlled clinical trial	20 10 OLA vs 10 placebo	17.1 years 12–21 years	Anorexia nervosa	Olanzapine 10 mg/d	10 weeks	No significant difference in weight between the two groups Poorer tolerance of olanzapine treatment with elevated blood glucose and insulin
**La Via et al.**	1999	USA	Case report	2	15 and 27 years	Anorexia nervosa with purging behavior	Olanzapine 15 mg/d and 10 mg/d	14 weeks in hospital, then 6 months// 22 days in hospital, then 4 months	Improved adherence Reduced agitation Reduction in resistant ED symptoms with weight gain Discharge from hospital
**Leggero et al.**	2010	Italy	Uncontrolled nonrandomized open-label clinical trial	13	13.7 years	Anorexia nervosa	Olanzapine 4.13 mg/d	6 months	Significant difference in weight Improved global functioning Reduction in food-related symptoms Diminution in anxiety/depression symptoms Diminution of hyperactivity
**Malina et al.**	2003	USA	Retrospective study	18	22 years	Anorexia nervosa	Olanzapine 4.7 mg/d	17 weeks	Diminution of obsessive ideas focused on body image Reduced anxiety/fear around meals Better acceptance of weight gain Better sleep at night
**Mehler et al.**	2001	Germany	Case report	5	12–17 years	Anorexia nervosa	Olanzapine 5–12.5 mg/d	6 weeks// 8 weeks in hosp and 2 months postdischarge// 2 months// 7 weeks// Not reported	Reduction in food-related symptoms Best adherence with proposed care No weight gain
**Mondraty et al.**	2005	Australia	Randomized controlled open-label clinical trial	15 8 OLA vs 7 chlor-promazine	25.3 years	Anorexia nervosa	Olanzapine 10 mg/d *(chlorpromazine = 50 mg/d)*	Between 46 and 53 days	No significant difference in weight between the two groups Diminution in anorexic cognitions in the olanzapine group
**Norris et al.**	2011	Canada	Retrospective study	86	10–17 years	Anorexia nervosa	Olanzapine 5 mg/d (median dose)	252 days	Conclusion about effectiveness impossible Group treated by olanzapine more severely ill than control group Surveillance of tolerability with monitoring of laboratory indicators
**Pisano et al.**	2014	Italy	Case report	5	13 years	Anorexia nervosa Bulimia (1)	Olanzapine 2.5 mg 2.5–7.5 mg/d	6 months	Increased calorie intake and weight gain Reduction in food-related symptoms
**Powers et al.**	2001	USA	Uncontrolled nonrandomized open-label clinical trial	20	14–56 years	Anorexia nervosa	Olanzapine 10 mg/d	10 weeks	Significant difference in weight Good tolerance with sedation as the most frequent adverse effect
**Pruccoli et al.**	2022	Italy	Retrospective study	118 37 OLA low dose 29 OLA full dose 52 controls	15.4 years	Anorexia nervosa	Olanzapine ≤5 mg/d (low dose) Or Olanzapine >5 mg/d (full dose) Or No SGA	5 months	Low-dose olanzapine was tolerable and safe. Greater improvements in depressive symptoms with low-dose olanzapine, or without antipsychotic, than with full-dose olanzapine.
**Spettigue et al.**	2018	Canada	Nonrandomized open-label clinical trial	32 14 OLA vs 10 in the control group	11–17 years	Anorexia nervosa	Olanzapine 2.5–15 mg/d	12 weeks	No significant difference in weight between the two groups Good clinical tolerance, plus side effects in the olanzapine group

∇ Clinical Trials

*Randomized controlled trials*

The first randomized controlled clinical trial (RCTs) took place in 2005, and most between 2007 and 2011. Overall, 7 clinical trials studying 294 AN patients have been published. The first tested olanzapine against chlorpromazine, while the other six used placebo controls. The olanzapine doses varied from 2.5 to 10 mg/d, with a mean 11-week follow-up. Most took place in a general population of young adults, with only one in the general population of adolescents.

Five RCTs (Mondraty [26], Brambilla [27], Brambilla [28], Bissada [29], Kafantaris [30]) did not report a significant difference in weight between the olanzapine group and the control groups. Only the two most recent studies, both by the team of Attia et al. [31,32], observed a significant increase in BMI after olanzapine treatment began.

Four studies also examined its tolerance profile. Brambilla et al. [27,28], Bissada et al. [29] and Attia et al. [31,32] reported no major adverse effects. The most frequent side effect was sedation. Kafantaris et al. [30] warned of a risk of elevated blood glucose and blood insulin rates in the general pediatric population, while Attia et al. [31] found no modification of glucose or lipid levels in the general adult population.

*Uncontrolled nonrandomized trials*

The literature includes four uncontrolled, nonrandomized clinical trials. All focused on an adolescent or young adult population. All four reported a significant improvement in weight after olanzapine treatment.

In 2001, Powers et al. [33] were the first to publish an open-label clinical trial with no control group. The authors observed a significant difference in weight in patients and good clinical tolerance; sedation was the principal side effect reported. Three years later, Barbarich et al. [34] confirmed these first results by Powers et al. [33]. They also observed a significant difference in weight and a reduction in ED symptoms.

Leggero et al. [35] and Spettigue et al. [36] conducted clinical trials in the general adolescent population. Both teams concluded that weight improved significantly and that clinical tolerance was good. Leggero’s team [35] also reported an improvement in global functioning and a reduction in symptoms of mixed anxiety and depression and of hyperactivity.

∇ Retrospective studies

Between 2000 and 2022, only five retrospective studies assessed the effectiveness of olanzapine in patients treated for AN. Three took place in general adult populations, and two in general adolescent populations.

In 2003, Malina et al. [37] published a retrospective study of the effects of olanzapine in 18 women treated for AN. The results showed a reduction in both obsessive ideas focused on body image and in fear of meals, as well as better acceptance of food intake. The women reported that they accepted weight gain better, felt more at ease in stressful situations, and slept better at night. Bosanac et al. [38] and Himmerich et al. [39] both reported weight gains after patients began olanzapine. In 2022, the last retrospective study [40] conducted on 118 adolescents showed individuals treated with low-dose olanzapine (≤ 5 mg/d) and those treated without any SGA reported better outcomes on depressive symptoms than those treated with full-dose olanzapine (> 5 mg/d). Furthermore, in the subsample receiving low-dose olanzapine, the treatment was safe and well tolerated. In terms of tolerability, Himmerich et al. [39] reported few changes in laboratory values. Laboratory monitoring, especially of the lipid profile, is recommended, as suggested by Norris et al. [41]. These adverse drug reactions were not confirmed in the study by Pruccoli et al. [40].

These retrospective studies suggest that olanzapine has a beneficial effect on weight and ED symptoms. Nonetheless the performance of methodical, rigorous, and standardized clinical trials is encouraged to corroborate these first results.

∇ Case reports

Since the beginning of the century, eight case reports (references 39–46) have described the effectiveness of olanzapine in patients with AN. Two case reports concerned a total of 4 adult women, while six described 23 adolescent girls, all treated by olanzapine for AN.

- Only one case report did not observe weight gain [42], while the others (La Via [43], Jensen [44], Boachie [45], Ercan [46], and Pisano [47]) all reported that the patients gained weight.
- Several authors described a diminution in ED symptoms, dysmorphic disorder, and anorexic cognitions. Similarly, improvement in psychiatric symptoms has been reported, with a reduction in anxiety, agitation, and sleep disorders [48].
- Ercan [46] and Duvvuri [49] each described a cases of recovery from eating disorders.

It should be noted that good clinical tolerability was observed in the general pediatric population, except for two girls who stopped the treatment because of excessive sedation.

Overall, these different case reports from pediatric and adult populations have principally emphasized weight gain and a reduction in anorexic cognitions and mixed anxiety and depression symptoms. Some authors [47] have proposed eligibility criteria for a prescription of olanzapine: for patients with no response at the end of a month of psychotherapy, food intake less than 800 kcal/d, associated mood disorders that are clinically severe, with major functional repercussions and massive denial.

Conclusion: The results of the randomized controlled clinical trials on the effect of olanzapine on weight vary between studies (Table 1). This may be explained by different methodologies and heterogeneous populations. The two systematic reviews indicate evidence of a beneficial effect by olanzapine in some specific situations. Most of the articles indicate that the tolerance profile in this population is good.

#### b) Aripiprazole

Aripiprazole is different from the other SGA. It is, among other things, a partial agonist of the dopamine D2 and serotonin 1A and 2C receptors, as well as an antagonist of the serotonin 2A receptor [24].

∇ Clinical trials:

Our search found no clinical trials—RCTs or other—that specifically studied the effect of aripiprazole in AN.

∇ Retrospective studies

Two retrospective studies, published in 2017 by Frank et al. [50] and in 2020 by Tahıllıoğlu et al. [51], assessed a total of 33 adolescents treated for AN by ariprazole. Both teams reported significant weight gains. Like the retrospective studies discussed above (50,51), they observed a reduction in cognitive rigidity and in obsessive ideas focused on food (calorie counting and dietary selectivity) and weight. Aripiprazole appeared to permit improved acceptance of weight gain. Tahıllıoğlu et al. [51] consider that it is better tolerated than other SGAs, with few side effects reported. Nonetheless these studies have several limitations: they are retrospective, have no control group, small sample sizes, come from single centers, and involve patients prescribed concomitant medications.

∇ Case series

In 2011, M.E. Trunko’s team [52] published a case series describing eight women with AN or anorexia of the bingeing/purging type treated with aripiprazole; in 2016 G.K.W. Frank published a case report about four adolescents [53]. Their observations provide some support for a beneficial effect by aripiprazole on anxiety as well as a reduction in obsessive ideas focused on food and anorexic cognitions. Both underline better acceptance of weight gain associated with a diminution of the body dysmorphic disorder. Trunko et al. [52] observed improvements in cognitive flexibility, and Frank’s team reported improved insight that enabled a better investment in treatment. Finally, the benefit may be increased when associated with an SSRI such as fluoxetine.

In terms of tolerability, Frank [53] noted the risk of neutropenia and the importance of regular monitoring with laboratory tests.

Conclusion: Case series and retrospective studies account for most of the literature about aripiprazole (Table 2). No clinical trial has been reported. These first studies offer support for a positive effect on cognitive rigidity and obsessive ideas focused on food and weight.

**Table 2:** Articles included in the scoping review concerning aripiprazole, quetiapine, and risperidone.

Study	Year	Country	Study method	Number of subjects	Mean age and/or range	Type of ED	AAP + dose and/or mean dose	Study duration	Results
**Bosanac et al.**	2007	Australia	Open-label clinical trial	7	MA = 33.5 years	Anorexia nervosa) Anorexia nervosa with bingeing-purging behavior (4)	Quetiapine 50–800 mg/d	8 weeks	Good clinical tolerance for quetiapine, no major adverse event Positive effect on ED symptoms and weight
**Carver**	2001	USA	Retrospective study	30		Resistant anorexia nervosa	Risperidone 0.5–1.5 mg/d		Weight gain Increased calorie intake
**Court et al.**	2010	Australia	Randomized controlled clinical trial	33 (1♂) 15 quetiapine vs 18 control group	MA = 23.8 years 15–42 years	Anorexia nervosa	Quetiapine 50–400 mg/d	12 weeks	Good clinical tolerance of treatment by quetiapine, no major adverse event observed Possible beneficial effect of small doses of quetiapine
**Frank et al.**	2016	USA	Case report	4	12, 12, 12, and 17 years	Anorexia nervosa (2) Anorexia nervosa with purging behavior (2)	Aripiprazole 2–5 mg/d	≈ 1 year	Reduction in anxiety and anorexic cognitions Improved insight Reduction in fear of weight gain and of body dysmorphic disorder
**Frank et al.**	2017	USA	Retrospective study	106 22 aripiprazole vs 84 control group	MA = 14.4 years	Anorexia nervosa	Aripiprazole 1–5 mg/d	Between 50 and 60 days	Possible beneficial effect on weight Improvement of rigid anorexic cognitions Improvement of weight restoration process
**Hagman et al.**	2011	USA	Double-blinded randomized controlled clinical trial	40 18 risperidone vs 22 placebo	MA = 16 years 12–21 years	Anorexia nervosa	Risperidone 0.5–4 mg/d DM risperidone 2.5 mg/d	11 weeks	No significant difference in ED symptoms No significant difference in weight Good tolerance of the treatment by risperidone
**Kracke & Tosh**	2014	USA	Case report	1	17 years	Anorexia nervosa	Risperidone 0.5 mg/d	4 years	Weight gain Reduced rigidity at meals Resumption of menstrual cycles
**Mehler-Wex et al.**	2008	Germany	Case report	3	11, 14, and 15 years	Anorexia nervosa (1) Anorexia nervosa with purging behavior (1) Anorexia nervosa with hyperactivity (1)	Quetiapine 200–500 mg/d	3–6 months	Diminution of internal tension Improved mood and mood stability Reductive of restrictive symptoms Better acceptance of weight gain
**Newman-Toker**	2000	USA	Case report	2	12 and 19 years	Anorexia nervosa	Risperidone 1.5 mg/d		Reduction of anxiety Normalization and stabilization of weight Improved mood Improved insight
**Powers et al.**	2007	USA	Pilot open-label clinical trial	20	MA = 26.8 years 14–48 years	Anorexia nervosa (12) Anorexia nervosa with bingeing-purging behavior (8)	Quetiapine 150–300 mg/d	10 weeks	Significant reduction in PANNSS scores Diminution in anxiety/depression symptoms Good clinical safety with mild side effects No significant difference in weight
**Powers et al.**	2012	USA	Double-blinded randomized controlled clinical trial	15 (1♂) 6 quetiapine vs 9 placebo	34 years	Anorexia nervosa (8) Anorexia nervosa with bingeing-purging behavior (7)	Quetiapine 177.7 mg/d	8 weeks	No significant difference of ED symptoms between the placebo group and the quetiapine group
**Tahıllıoğlu et al.**	2020	Turkey	Retrospective study	11	11–17 years	Anorexia nervosa	Aripiprazole 2.5–15 mg/d	20–28 months	Significant improvement in obsessive symptoms Significant weight gain Better clinical tolerance than the other AAPs
**Trunko et al.**	2011	USA	Case report	8	15, 21, 28, 30, 33, 35, 52, and 55 years	Anorexia nervosa (5) Bulimia (3)	Aripiprazole 5–15 mg/d	4 months–3 years	Reduced anxiety about food, weight, and body image Better acceptance of weight gain Better cognitive flexibility
**Umehara et al.**	2014	Japan	Case report	1	10 years	Anorexia nervosa (1♂)	Risperidone 1 mg/d 12.5 mg/2 weeks of long-acting risperidone	3 years	Resumption of oral eating Regression of EDs Weight gain

#### c) Quetiapine

Quetiapine is, among other things, a relatively weak antagonist of the dopamine D1 and D2 and the serotonin 1A and 2A receptors. It is also a strong antagonist of histamine H1 receptors [24].

∇ Clinical trials

Four clinical trials were published between 2007 and 2022 to assess quetiapine in AN. Most of these were pilot studies that assessed its tolerability and safety for this indication. Two studies (Bosanac et al. [54], Court et al. [55]) report good tolerance with no major adverse events. According to Court et al. [55], the side effects most frequently reported are fatigue, sedation, concentration problems, and orthostatic vertigo. Most of these start with the treatment but are transient.

In 2001, Powers et al. [33] found that a modest weight gain with olanzapine was accompanied by statistically significant decreases in total scores on the Positive and Negative Syndrome Scale (PANSS). In 2007, the same team [56] evaluated the effectiveness of quetiapine in AN with the PANSS. They observed a significant drop in scores after the initiation of quetiapine at doses between 150 and 300 mg/d, but weight gain was not significant. In 2012, Powers et al. [57] evaluated the effectiveness of quetiapine in AN with a double-blinded placebo-controlled trial. The PANSS scores showed no statistically significant differences between the quetiapine and placebo groups, and the authors did not document any significant difference in anorexic symptoms between the two groups.

In 2007, Bosanac’s team [54] conducted an open-label study. It concluded that quetiapine had a positive effect on weight and diminished ED symptoms over 8 weeks.

These studies present numerous biases. Most encountered problems during the study with either with recruitment or a high dropout rate; these explain their small sample sizes. Two studies (Powers et al. [56] and Bosanac et al. [54] were open-label and had no control group. The duration of follow-up was relatively short (from 8 to 12 weeks).

∇ Retrospectives studies

Our search found no retrospectives studies that specifically studied the effect of quetiapine in AN.

∇ Case reports

In 2008, Mehler-Wex et al. [58] published one case report about three girls aged 11, 14, and 15 years treated for AN in whom the authors observed mood improvement and stabilization after quetiapine treatment began. They also observed weight gain and stabilization associated with a reduction in anorexic cognitions and restrictive behaviors, as well as a reduction in physical distortions and, for one woman, better acceptance of weight gain. They also noted a decrease of anxiety in two women. The treatment was well tolerated clinically. The authors underlined that the treatment by quetiapine was less likely than other medications to induce weight gain. The effect may be indirect and may not be that sought in priority; this may diminish patients’ reluctance and enable better adherence.

Conclusion: Most of the literature about the prescription of quetiapine in AN (Table 2) comes from case reports and clinical trials that were mostly open-label, with small sample sizes and short follow-ups. The results are consistent with a positive effect on mood. The positive effect on weight may result from improvement of mood and ED symptoms. They underline the good clinical tolerance in this population.

#### d) Risperidone

Risperidone is a powerful dopamine D2 antagonist, especially at high doses. It also acts as an antagonist for the serotonin 1A and 2A and histamine H1 receptors [24].

∇ Clinical trials

Hagman et al. [59] published the first and only randomized double-blinded, placebo-controlled trial to assess the prescription of risperidone in adolescents and young adults treated for AN. This exploratory pilot study sought to assess the efficacy and safety of risperidone for AN. The authors hypothesized that the participants receiving risperidone would undergo a significant diminution in their drive for thinness, their feelings of body dissatisfaction, and in their body image distortions compared with participants receiving the placebo. The study did not show a significant difference between the two groups and did not demonstrate any net benefit from the use of risperidone. Its principal limitation was its major difficulty in recruitment; it turned out to be impossible to reach the objective of including 50 patients. The team nonetheless noted that the risperidone treatment was well tolerated in this population.

∇ Retrospective study

In 2002, Carver [60] published a retrospective study of 30 patients who received a prescription for risperidone at a dose of 0.5–1.5 mg/d. The group treated by risperidone gained more weight and increased their daily calorie intake more.

∇ Case reports

Since the beginning of the century, three case reports—by Newman-Toker [61], Kracke and Tosh [62], and Umehara et al. [63]—have assessed the prescription of risperidone in four women with anorexia. All the teams noted a weight gain associated with the resumption of oral feeding or an increase in calorie intake. Newman-Toker [61] also observed a reduction in anxiety, better mood, and improved insight. The reports by Newman-Toker [61] and Kracke and Tosh (Kracke & Tosh, 2014) suggested a diminution of cognitive rigidity and obsessive ideas. Umehara et al. [63] underlined the interest of the injectable form of risperidone.

Conclusion: The literature about the prescription of risperidone in AN (Table 2) is very sparse and its results contradictory. It comprises several case reports, one retrospective study, and only one clinical trial. The case reports and retrospective study are consistent with a beneficial effect not only on weight but also on anorexic cognitions and mood. The only randomized controlled trial did not confirm these clinical observations; it found no significant differences between the groups.

#### e) Literature reviews and meta-analyses on the use of SGAs in AN

Between 2005 and 2022, six systematic reviews of the literature and four meta-analyses assessed the prescription of antipsychotics in AN. Olanzapine was the substance assessed most often, accounting for 66% of the reports.

The first review of the literature [64] comprised principally case reports, retrospective studies, and open-label clinical trials without a control group and with small sample sizes and short follow-ups. These characteristics made it difficult to reach conclusions or establish recommendations. In 2008, Mehler-Wex et al. [58] concluded that SGAs are indicated in severe cases resistant to first-line treatment, with an extreme fear of gaining weight, with either an invasive body dysmorphic disorder or disabling physical hyperactivity. McKnight and Park [65] considered that the evidence suggests a beneficial effect by SGAs against ED symptoms, anxiety, and depressive symptoms.

Three meta-analyses—by Lebow et al. [15], Kishi et al. [14], and Dold et al. [66]—of RCTs assessing the prescription of SGAs in the adult general population failed to confirm these initial results and concluded that SGAs do not significantly affect weight gain. Furthermore, Lebow et al. [15] and Kishi et al. [14] did not observe any positive effect on anorexic cognitions. Lebow et al. [15] also warned about the risk of increased anxiety. However, a meta-analysis from 2022 [67], including 304 patients with AN, showed that the adults in the olanzapine group had a significant increase in BMI compared with those in the placebo group. No significant difference was observed in adolescents, although olanzapine as adjuvant treatment showed a trend toward improved BMI. In the first review of SGA prescriptions in children and adolescents, Couturier et al. [68] judged that the evidence was not sufficient to recommend these drugs as a first-line treatment in this population.

From the perspective of tolerability, Mehler-Wex et al. [58] recommended low-dose prescriptions for children and regular monitoring.

The systematic review of the literature published by Dunican et al. [69] studied only the role of olanzapine in the treatment of AN. In this article the authors relied principally on case reports and retrospective studies, with no RCTs. According to them, olanzapine can be useful in regaining and stabilizing weight as well as in the diminution of anorexic symptoms. In 2020, Çöpür et al. [70] published a new systematic review of the literature concerning olanzapine prescription that this time considered more studies, including five RCTS. These authors encouraged the use of olanzapine for the treatment of AN and underlined the benefit of prescribing an olanzapine dose > 5 mg/d for a relatively short duration (< 8 weeks).

All of these systematic reviews of the literature and meta-analyses (Table 3) agreed that better quality clinical trials are required to establish more robust scientific evidence. Couturier et al. [68] pointed out the issues of recruiting patients and keeping them from dropping out of these studies. The authors also note the importance of organizing several sites to collaborate on multicenter trials that can provide high-quality evidence to enable the establishment of more robust guidelines.

**Table 3:** Literature reviews and meta-analyses of atypical antipsychotic drugs in AN included in the scoping review.

Study	Year	Country	Study method	Study size	Types of studies included	Treatments studied	Results
**Bosanac et al.**	2005	Australia	Systematic review of literature	7	5 open-label clinical trials 1 single-blinded trial 1 randomized clinical trial	Olanzapine (4), risperidone (2), and amisulpride (1)	No conclusion possible because of the absence of any randomized placebo-controlled clinical trial
**Çöpür &Çöpür**	2020	Turkey	Systematic review of the literature	24	**5 randomized controlled trials** 8 case-control studies 11 case reports	Olanzapine	Possible benefit on weight of olanzapine > 5 mg/d during < 8 weeks
**Couturier et al.**	2019	Canada	Systematic review of the literature	19	4 controlled trials 3 open-label trials 2 retrospective studies 10 case reports	Olanzapine (12), risperidone (2), quetiapine (2), and aripiprazole (3)	Insufficient scientific evidence to recommend the prescription of second generation antipsychotics in the general pediatric population
**Dold et al.**	2015	Austria	Meta-analysis	7	**7** randomized controlled trials	Olanzapine (4), quetiapine (2), and risperidone (A)	No significant difference for weight gain
**Dunican & DelDotto**	2007	USA	Systematic review of the literature	12	8 case reports 2 prospective studies 2 retrospective studies	Olanzapine	Olanzapine can be useful in regaining and stabilizing weight as well as in the diminution of anorexic symptoms
**Han et al.**	2022	Japan	Meta-analysis	7	5 randomized controlled trials 1 retrospective study 1 open label clinical trial	Olanzapine	Efficacy of olanzapine in the treatment of AN, with weight gain at the end of treatment in adults. Effect unclear in adolescents.
**Kishi et al.**	2012	USA	Meta-analysis	8	8 randomized controlled trials against placebo or usual care	Olanzapine (4), quetiapine (1), risperidone (1), and sulpiride (2)	No significant difference for weight gain No effects on anorexia symptoms
**Lebow et al.**	2012	USA	Meta-analysis	8	8 randomized controlled trials	Olanzapine (6), risperidone (1), and amisulpride (1)	No significant difference in weight No effect on anorexic cognitions Possible increase in anxiety Possible reduction in depressive symptoms
**McKnight & Park**	2010	UK	Systematic review of the literature	36	26 case studies 6 open-label or single-blinded trials 4 randomized controlled trials	Olanzapine (26), quetiapine (6), aripiprazole (6), risperidone, and amisulpride	Possible reduction in food-related symptoms, anxiety, and depressive symptoms Very modest effect on weight
**Mehler-Wex et al.**	2008	Germany	Systematic review of the literature	15	9 case reports 1 retrospective study 4 open-label clinical trials 1 randomized clinical trial	Olanzapine (10), risperidone (2), and quetiapine (2)	Indication for AAPs in severe cases resistant to first-line treatment, with an extreme fear of gaining weight, an invasive body dysmorphic disorder or physical hyperactivity

### II. Tolerability of SGA in the management of AN

Seven articles focused on the tolerability of SGA in this vulnerable clinical population exposed to numerous somatic complications linked to malnutrition. Two articles looked at disorders of carbohydrate metabolism, one each at other metabolic effects, cardiac disorders, and diverse reported side effects, and two more studied neurological effects with neuroleptic malignant syndrome and somnambulism.

The articles about carbohydrate metabolism noted two types of abnormalities:

- Yasuhara et al. [71] observed the appearance of diabetes in a young (27-year-old) Japanese woman with AN treated by olanzapine 5 mg/d, perhaps involving glucose intolerance combined with insulin resistance. The authors observed an improvement in blood glucose levels following the normalization of her weight.
- Inversely Haruta et al. [72] published a description of episodes of severe hypoglycemia in a 34-year-old Japanese woman treated with paroxetine and olanzapine. The specific mechanism is not known, but the hypoglycemia may be associated with insulin hypersecretion in women with reduced glucose stores [73]. Several reports have noted that paroxetine can cause hypoglycemic episodes The co-prescription of olanzapine and paroxetine therefore requires increased vigilance.

Kan et al. [74] published a meta-analysis assessing the dropout rate and metabolic effects associated with antipsychotic prescriptions in AN. The authors remarked that patient-associated factors explain dropout rates in the trials more than drug-related side effects do. The data about metabolic effects are heterogeneous, even contradictory. Some studies report weight gains, while others do not. Similarly, the data about carbohydrate metabolism and cardiac conduction are difficult to interpret and do not authorize any conclusions. Most of the studies did not show a significant difference in lipid profiles, liver function, or prolactin levels.

Kan et al. [74] also examined, as did Swenne et al. [75], diverse side effects associated with the prescription of olanzapine. Kan’s team [74] reported that sedation is the most frequently reported adverse effect, followed by vertigo, headaches, gastrointestinal problems, insomnia, fatigue, and muscle pain. Some studies mention dry mouth, concentration issues, vision problems, or even paresthesia, but Kan et al. did not report prevalence rates. Swenne et al [75] published the cases of three adolescents (aged 12, 15, and 17 years) who had stopped treatment after major adverse events associated with their olanzapine prescriptions: elevated liver enzymes hepatic four weeks after the start of treatment, two epilepsy seizures in an adolescent with no such history, and the onset of episodes of galactorrhea and breast sweats. Apart from these three major events, most of the adolescents experienced only mild adverse effects. In three, the introduction of olanzapine was accompanied by TSH disorders and the discovery of autoimmune thyroid diseases, but no cause-and-effect relation was established.

From the cardiac point of view, Ritchie and Norris [76] published the case of a 15-year-old girl with a QTc prolongation observed after she began SGA treatment by olanzapine and then risperidone. A switch to quetiapine led to a correction of the QT and subsequent clinical improvement. The authors recommend the performance of closely-spaced ECGs when this treatment is begun, and dose modification in malnourished patients at risk of complications. They insist on the need for particular vigilance in women with purging behavior, in whom electrolyte disorders can accentuate cardiac conduction disorders.

Ayyıldız et al. [77] published a neurological adverse event: a neuroleptic malignant syndrome diagnosed in a 17-year-old youth two days after he started treatment by olanzapine at a dose of 5 mg/d; he required transfer to intensive care. Stopping the olanzapine treatment corrected the symptoms. Recently, de Filipis et al [78] reported a first case of somnambulism induced by olanzapine in ED. More pharmacovigilance studies are required.

Conclusion: From the perspective of tolerability, SGAs have a fairly good profile in this population at particular somatic risk. The frequent side effects are mainly mild and transient. Most patients who stopped treatment did not do so due to an adverse effect of the drug. The authors nonetheless insist on the need for regular clinical and laboratory monitoring. They encourage carbohydrate and lipid monitoring as well as regular ECGs.

## Discussion

This review describes the results of 55 studies, 48 assessing the effectiveness of SGAs in AN and 7 focusing on their tolerability and safety in this population at particular somatic risk. Olanzapine is the SGA most often studied in the scientific literature; the primary outcome measure of these studies is generally weight. Most of the RCTs (5 of 7 studies) did not observe a significant modification of weight. The other SGAs have been evaluated much less often, with very few clinical trials: 4 for quetiapine but only 1 for risperidone and none for aripiprazole. In view of the contradictory results and often modest effects observed, the reviews of the literature remain prudent in their conclusions and do not recommend using SGAs as a first-line treatment for AN.

The tolerability of these four medications was globally good in this population at risk. The side effects are mostly mild and transient [74]. These prescriptions nonetheless necessitate close cardiac, laboratory, and neurological monitoring [76,77].

Olanzapine is the psychotropic medication most frequently prescribed for AN [79], ahead of antidepressant treatments such as fluoxetine. SGAs are therefore regularly used in clinical practice for EDs. Fazeli et al. [80] published a study assessing the use of psychotropic medications for treating AN in the USA between 1997 and 2009. They observed that the use of antipsychotics had doubled in less than 10 years and that around 13% of patients with AN received antipsychotic treatment, most often an SGA. In 2020, Muratore and Attia [79] found concordant prescription rates in England. These studies thus estimate that around one patient with anorexia in 10 receives an antipsychotic treatment. Nonetheless, as we have seen in this scoping review, the studies assessing their effectiveness in AN are sparse, have low power, and have produced contradictory results. These heterogeneous findings are also reflected in the international guidelines, where the place of SGAs is unclear and varies from country to country. Some countries, such as France [81], only mention SGAs in their AN management decision tree to warn about the risk of side effects and do not describe their utility. Others, such as the USA [82] and the UK [83], are prudent and indicate that the level of evidence for these treatments is limited because of the numerous methodological limitations of the various studies. Inversely, some countries encourage the use of olanzapine. One is Canada [84], which states in its most recent guidelines that the use of olanzapine and aripiprazole can be envisioned as adjuvant treatment for children and adolescents with AN and severe weight loss. The same is true for Australia [85], which indicates that olanzapine can be useful especially to diminish hyperactivity and reduce anorexic cognitions.

This absence of international consensus together with the heterogeneous and contradictory results raises questions: why have the different studies thus far conducted not allowed a clear conclusion about the effectiveness—or ineffectiveness—of SGAs in AN? All of the guidelines agree that the studies thus far conducted have numerous limitations that reduce their statistical power and their robustness.

The first limitations are linked to the difficulty of setting up large homogeneous samples with few patients lost to follow-up. The studies analyzed in this scoping review all faced recruitment problems. Several studies did not reach the number of participants initially planned, while others had to prolong their recruitment periods to obtain the necessary number of participants. As the AACAP Research Forum has noted [86], “poor recruitment is the principal factor limiting the success of numerous trials and surveys.” Norris et al. [87] looked at the factors that decrease research participation rates in this specific population. The lack of insight in most of these patients leads to their ambivalence about care and their reluctance to receive treatment or participate in research programs. The fear of potential side effects is also a reason for refusal. In their study of 27 eligible women, 20 refused to participate, for a 74% refusal rate: 55% of the refusals came from patients, 45% from their families.

These studies also face numerous losses to follow-up. Halmi et al. [88] published a prospective study in 2005, assessing the factors leading to the unacceptability of and failure to complete treatment for two specific therapies for AN: pharmaceutical treatment by antidepressants and cognitive behavioral therapy. The authors observed that the attrition rate at the beginning of treatment was particularly high in the medication arm. This suggests that some participants who had initially indicated a willingness to receive drugs and/or psychotherapy were in fact not willing to envision pharmacotherapy alone and preferred to drop out of the study. The acceptability and long-term adherence to pharmacological treatment is therefore a challenge in this specific population.

These problems with recruitment and retention in trials are combined with the issue of putting together homogeneous samples. The variety of clinical pictures and patient profiles makes it all the more difficult to interpret results and generalize them in clinical practice. A recent systematic scoping review [89] of treatments for major depressive disorder in adolescents with AN reached the same conclusion. In that study, the authors recommended a better definition of the subtypes of AN and the phenotypic characteristics of the patients included (age, sex, pubertal stage at AN onset, etc.) to make these studies reproducible and enable psychiatrists to improve our therapeutic conduct.

Study design is thus an important question. Quantitative studies of the type considered for evidence-based medicine do not appear capable of responding fully to the questions asked. It appears to us that it would be interesting to conduct innovative studies that are different from those performed in the past. Qualitative studies could make it possible to overcome the obstacles encountered up to now. They are an example that benefits from, rather than being limited by, the quasi-systematic heterogeneity of samples. This type of study could make it possible to open up pathways of reflection and bring to light perspectives barely if at all envisioned until now. In focusing on the viewpoints of patients and their close family and friends, these studies could make it possible to better represent what they consider "helpful" and to adjust treatment propositions accordingly. Qualitative studies are becoming increasingly common and have already had an impact on current management strategies [90].

But the difficulties of recruitment, sampling, and design are not the only limitations of these studies. In 2009, Crow et al. [91] published an article shedding light on other reasons that limit the conclusions of these trials. They explained the lack of proof of these studies’ effectiveness by their choice of a poor principal endpoint. That is, weight is the outcome measure chosen most frequently in studies to assess the effectiveness of SGA treatment in AN. Nonetheless, this criterion presents several limitations. First, individuals with AN have an inordinate fear of gaining weight. Proposing a treatment intended, among other things, to make the patient gain weight has a very strong chance of being unacceptable to and thus of both preventing their participation in RCTs and increasing the likelihood of poor adherence. This objective thus does not appear to be a relevant strategy for care of AN, especially as this treatment is often prescribed in clinical practice for reasons other than weight, such as to combat hyperactivity or strong emotional disruption [79]. It may therefore be more appropriate to assess the effectiveness of these treatments on associated symptoms such as anxiety, cognitive rigidity, obsessive ideas, or perfectionism, or in facilitating refeeding and adherence to care rather than to hope to have a direct effect on eating and weight. Second, the patients, their families, and their healthcare providers all have different criteria for disease remission. Rance et al. [92] published a study that looked at the point of view of AN patients about the treatments for their diseases. The results showed a high degree of dissatisfaction with these treatments. Patients had the impression that treatments focused on ED symptoms and weight are not helpful and even considered that food- and weight-centered treatments tended to reinforce anorexic cognitions and the disease. They wanted the treatment teams to concentrate on other symptoms but also on the underlying cause ("Eating disorders are not about food, they’re about life"). This is also true for parents. Accurso et al. [93] published a study looking at parents’ points of view about their child’s recovery. Parents consider that it is important for their children to "engag[e] fully in social and professional activities" and thus “to return to normal functioning,” which includes emotional well-being and not only normal eating behaviors. These results should encourage researchers to move to a more holistic approach.

In addition to the difficulties associated with the study population and the study designs, there is probably an obstacle due to the drug class itself. It should not be forgotten that in our society antipsychotic treatments are principally associated with delusional disorders such as schizophrenia. These treatments with their negative connotations often frighten patients and their families, and they may find the proposal of a treatment commonly associated with "insanity" very upsetting. Antipsychotic drugs would probably benefit from being presented by a name underlining their mechanism, for example, “dopaminergic transmission modulators.” That would avoid confusion and the ensuing rejection.

Moreover, although AN is not considered a delusional disorder, these two disorders probably have similar underlying mechanisms. In clinical practice it is common to observe similar symptoms, and the correct diagnosis can be unclear. The categorical approach thus appears to have its limitations. Relatedly, Behar et al. [4] published a literature review that assessed the delusional aspects of AN. They argue that the distinction between delusions and obsessive ideas can be difficult in this disorder. The study by Powers et al. [56], using the PANSS (Positive and Negative Syndrome Scale) to assess the effectiveness of quetiapine in AN, also fits into this perspective. They observed a significant reduction in the scores of a scale used in schizophrenia, although these patients had not been diagnosed as psychotic (which was an exclusion criterion). Psychosis and AN may thus be more closely linked than it appears. Miotto et al. [94] published an article assessing psychotic symptoms in patients diagnosed with EDs and concluded that the frequency of these symptoms was higher in their sample of patients with EDs than in their large control sample of girls in good health. Behar et al. [4] reported psychotic episodes in 10–15% of women with EDs, while Foulon [95] affirmed that it can be difficult to recognize schizophrenia in patients with EDs. Their survey found a prevalence of schizophrenia of 3–10% in samples of patients with EDs. These studies thus raise questions about the overlap between diagnoses of psychosis and AN and about the potential benefits of SGA prescriptions in these clinical situations.

Our study has several limitations. We included only articles published in French or in English. Nonetheless, we found no articles published in French on this subject, and we excluded only three articles in a foreign language. Nearly all of the articles were thus published in English by authors from English-speaking countries. We included only articles published in this century. This choice is consistent with the historic data, since this is the period when SGA prescriptions began proliferating and research on their use in AN began.

## Conclusion

In conclusion, the studies assessing the effectiveness and tolerability of SGA have many limitations. The methodological difficulties they faced included long delays in recruitment, heterogeneous samples, and substantial numbers of patients lost to follow-up. The relevance of the principal endpoint chosen in these trials (most often, weight) is contested by some caregivers and most especially by patients, who would like to deemphasize the issues of weight and eating. Finally this drug class is associated with negative representations for patients and those close to them and thus negatively affects their participation in research and their adherence to these treatments. These limitations prevent the studies from reaching conclusive, reliable, robust, and reproducible results. Nonetheless, SGAs continue to be prescribed regularly in clinical practice. SGAs cannot be the only treatment for AN and must not be prescribed either in first line or alone [84]. But olanzapine and aripiprazole could be interesting in clinical practice, in carefully selected cases, principally among patients with severe illness resistant to several lines of treatments. That is, these substances could be useful in cases involving marked obsessiveness and invasive, rigid anorexic cognitions. They could also be helpful in cases of disabling physical hyperactivity. Quetiapine is also frequently prescribed in this indication and seems particularly interesting in the case of severe mood-related comorbidity or anxiety resistant to initial treatments.

## Supporting information

S1 Table Characteristics of excluded studies. (DOCX)

S1 File PRISMA-ScR-Fillable-Checklist. (DOCX)
