# Supplementary material for: Efficacy and tolerance of second-generation antipsychotics in anorexia nervosa: A systematic scoping review
Source: PLoS One. 2023 Mar 16;18(3):e0278189. doi: 10.1371/journal.pone.0278189 (PMC10019643; doi:10.1371/journal.pone.0278189)
Supplement: S1 Table — (DOCX) [file pone.0278189.s001.docx]

**S1 : Characteristics of excluded studies**

| 1 | Pharmacotherapy for anorexia nervosa and bulimia nervosa  Greetfeld et al, 2011 | Articles in a foreign language |
| --- | --- | --- |
| 2 | Atypical antipsychotics in child and adolescent psychiatry--indications apart from schizophrenia.  Mehler-Wex et al, 2005 | Articles in a foreign language |
| 3 | Olanzapine in the treatment of adolescents with anorexia nervosa.  Hein et al, 2010 | Articles in a foreign language |
| 4 | Pharmacotherapy of eating disorders.  Davis et al, 2017 | Articles and clinical trials not specific for atypical antipsychotics |
| 5 | Psychopharmacological advances in eating disorders.  Himmerich et al, 2018 | Articles and clinical trials not specific for atypical antipsychotics |
| 6 | Recent advances in therapies for eating disorders.  Davis LE, Attia E, 2019 | Articles and clinical trials not specific for atypical antipsychotics |
| 7 | Pharmacologic Treatment of Eating Disorders.  Crow SJ, 2019 | Articles and clinical trials not specific for atypical antipsychotics |
| 8 | Psychopharmacologic treatment of eating disorders: emerging findings.  McElroy et al, 2015 | Articles and clinical trials not specific for atypical antipsychotics |
| 9 | Anorexia nervosa.  Fitzpatrick KK, Lock J, 2011 | Articles and clinical trials not specific for atypical antipsychotics |
| 10 | Atypical antipsychotics as augmentation therapy in anorexia nervosa.  Marzola et al, 2015 | Articles and clinical trials not specific for atypical antipsychotics |
| 11 | Inpatient treatment for anorexia nervosa: a systematic review of randomized controlled trials.  Suárez-Pinilla et al, 2015 | Articles and clinical trials not specific for atypical antipsychotics |
| 12 | Biological therapies for eating disorders.  Mitchell et al, 2013 | Articles and clinical trials not specific for atypical antipsychotics |
| 13 | Psychotropic medications in adult and adolescent eating disorders: clinical practice versus evidence-based recommendations.  Garner et al, 2016 | Articles and clinical trials not specific for atypical antipsychotics |
| 14 | Evidence-based pharmacotherapy of eating disorders.  Flament et al, 2012 | Articles and clinical trials not specific for atypical antipsychotics |
| 15 | Towards the pharmacotherapy of eating disorders.  Pederson et al, 2003 | Articles and clinical trials not specific for atypical antipsychotics |
| 16 | Pharmacotherapy for eating disorders and obesity.  Powers et al, 2009 | Articles and clinical trials not specific for atypical antipsychotics |
| 17 | Pharmacologic treatment of anorexia nervosa: where do we go from here?  Attia E, Schroeder L, 2005 | Articles and clinical trials not specific for atypical antipsychotics |
| 18 | What is the scientific evidence for the use of antipsychotic medication in anorexia nervosa?  Court et al, 2008 | Articles and clinical trials not specific for atypical antipsychotics |
| 19 | Survey on self-reported psychotropic drug prescribing practices of eating disorder psychiatrists for the treatment of young people with anorexia nervosa.  Y Beykloo et al, 2019 | Articles and clinical trials not specific for atypical antipsychotics |
| 20 | Current Therapeutic Approaches to Anorexia Nervosa: State of the Art.  Muratore et al, 2020 | Articles and clinical trials not specific for atypical antipsychotics |
| 21 | Available pharmacological treatments for anorexia nervosa.  Powers et al, 2004 | Articles and clinical trials not specific for atypical antipsychotics |
| 22 | Psychotropic drug treatment in anorexia nervosa. Search for differences in efficacy/tolerability between adolescent and mixed-age population  Matteo Balestrieri et al, 2013 | Articles and clinical trials not specific for atypical antipsychotics |
| 23 | Psychotropic medication use in anorexia nervosa between 1997 and 2009.  Fazeli et al, 2012 | Articles and clinical trials not specific for atypical antipsychotics |
| 24 | Drug therapy for patients with eating disorders.  Mitchell et al, 2003 | Articles and clinical trials not specific for atypical antipsychotics |
| 25 | Pharmacological treatment of eating disorders, comorbid mental health problems, malnutrition and physical health consequences  Himmerich et al, 2021 | Articles and clinical trials not specific for atypical antipsychotics |
| 26 | Current and emerging directions in the treatment of eating disorders.  Brown TA, Keel PK, 2012 | Articles and clinical trials not specific for atypical antipsychotics |
| 27 | A review of medication use for children and adolescents with eating disorders.  Couturier et al, 2007 | Articles and clinical trials not specific for atypical antipsychotics |
| 28 | Pharmacological treatment of acute-phase anorexia nervosa: Evidence from randomized controlled trials.  Cassioli et al, 2020 | Articles and clinical trials not specific for atypical antipsychotics |
| 29 | Current treatment for anorexia nervosa: efficacy, safety, and adherence.  Bodell et al, 2010 | Articles and clinical trials not specific for atypical antipsychotics |
| 30 | Drug Prescribing in Child and Adolescent Eating Disorder Services.  Gowers S et al, 2009 | Articles and clinical trials not specific for atypical antipsychotics |
| 31 | Psychotropic medication use at a private eating disorders treatment facility: A retrospective chart review and descriptive data analysis.  Gable KN, Dopheide JA, 2005 | Articles and clinical trials not specific for atypical antipsychotics |
| 32 | Psychotropic medication use in treatment-seeking youth with eating disorders.  Gorrell S et al, 2020 | Articles and clinical trials not specific for atypical antipsychotics |
| 33 | Medical management of eating disorders: an update.  Voderholzer et al, 2020 | Articles and clinical trials not specific for atypical antipsychotics |
| 34 | Tolerability and efficacy of aripiprazole in a case of psychotic anorexianervosa comorbid with epilepsy and chronic renal failure.  M Aragona, 2007 | Case reports with psychotic comorbidities |
| 35 | Olanzapine treatment in anorexia nervosa: case report.  Dadić-Hero et al, 2009 | Case reports with psychotic comorbidities |
| 36 | Evaluation of the efficacy and safety of olanzapine as an adjunctive treatment for anorexia nervosa in adolescent females: a randomized, double-blind, placebo-controlled trial.  Spettigue et al, 2008 | Unpublished results |
| 37 | Use of olanzapine in anorexia nervosa.  Menaster M, 2005 | Letter to the editor |
| 38 | Commentary to QTc prolongation associated with atypical antipsychotic use in the treatment of adolescent-onset anorexia nervosa.  Elbe D, 2009 | Letter to the editor, commentary |
| 39 | World Federation of Societies of Biological Psychiatry (WFSBP) guidelines for the pharmacological treatment of eating disorders.  Aigner et al, 2011 | International guidelines |
| 40 | Canadian practice guidelines for the treatment of children and adolescents with eating disorders.  Couturier et al, 2020 | International guidelines |
| 41 | Australian and New Zealand clinical practice guidelines for the treatment of anorexia nervosa.  Beumont P et al, 2004 | International guidelines |
